# Maternal childhood trauma and perinatal distress are related to infants’ focused attention from 6 to 18 months

**DOI:** 10.1038/s41598-021-03568-2

**Published:** 2021-12-17

**Authors:** Hsing-Fen Tu, Alkistis Skalkidou, Marcus Lindskog, Gustaf Gredebäck

**Affiliations:** 1 Department of Neurology, Max Planck Institute for Human Cognitive and Brain Sciences, 04103 Leipzig, Germany; 2 Department of Women’s and Children’s Health, Uppsala University, 75237 Uppsala, Sweden; 3 Department of Psychology, Uppsala University, 75237 Uppsala, Sweden

**Keywords:** Human behaviour, Psychiatric disorders, Psychology, Health care

## Abstract

Maternal distress is repeatedly reported to have negative impacts on the cognitive development in children and is linked to neurodevelopmental disorders (e.g. attention-deficit/hyperactivity disorder and autism spectrum disorder). However, studies examining the associations between maternal distress and the development of attention in infancy are few. This study investigated the longitudinal relationships between maternal distress (depressive symptoms, anxiety symptoms, and exposure to childhood trauma) and the development of focused attention in infancy in 118 mother-infant dyads. We found that maternal exposure to non-interpersonal traumatic events in childhood was associated with the less focused attention of the infants to audio-visual stimuli at 6, 10, and 18 months. In addition, exposure to interpersonal traumatic events in childhood was identified as a moderator of the negative effect of maternal anxiety during the 2nd trimester on the development of focused attention in infants. We discuss the possible mechanisms accounting for these cross-generational effects. Our findings underscore the importance of maternal mental health to the development of focused attention in infancy and address the need for early screening of maternal mental health during pregnancy.

## Introduction

Attention is a multifaceted construct and an important cognitive operation involving alerting, orienting, filtering, and attending to information in the environment^1,2^. The ability to focus and sustain attention develops rapidly in the first postnatal years^2–4^ and continues to develop into adulthood^5^ and has been postulated to play a fundamental role in learning^6–8^. More specifically, before the age of one, infants show learning behaviors through actively directing their attention to informative events and interacting with them^9,10^. Look duration, which is often used to measure focused attention, increases steadily from the second half of the first postpartum year and through the first four years of life^2,11^. To maintain focus for a period of time on tasks requires effort and hence it is often linked to the development of self-regulation and executive function in childhood^12–14^. There is substantial evidence showing that the ability to focus in infancy is predictive to social development^15^, cognitive functioning^16^, language development^17^, and academic skills^18^ later in life. In addition, poor focused attention is related to several neurodevelopmental disorders, such as attention-deficit/hyperactivity disorder (ADHD)^19^ and autism spectrum disorder (ASD)^20^. Taken together, identifying the risk factors of the development of focused attention in the early years is crucial for targeted prevention and early intervention.

Maternal psychological distress, which is symptomized by an unbalanced and/or strained emotional state from pregnancy to postpartum commonly including depression and/or anxiety^21,22^, is seen to influence the trajectories of attentional development in childhood. Several large cohort studies have shown that maternal depressive and/or anxiety symptoms are associated with attention problems in offspring at the ages of 2 years^23^, 3 and 4 years^24^, as well as 5, 6.5, and 14 years^25,26^. Maternal distress is also linked to ADHD symptoms at the age of 4 and 8–9 years^27,28^. Moreover, recent studies also reported that maternal childhood adverse experiences contribute to ADHD and ASD in children^29–31^. In nonhuman primates, exposure to mild stress during pregnancy is related to less visual exploration and higher distractibility of offspring^32^. In humans, maternal stress during pregnancy has a negative impact on infants’ attention shifting at the age of 18 months^33^. It is been shown that infants whose mothers perceived higher stress during pregnancy needed more time than others to process visual information at the age of 7.5 months and looked away from the tasks significantly more than infants whose mothers had low perceived stress during pregnancy^34^. Preliminary evidence also suggests that this cross-generational association between maternal distress and infant development might be linked to trauma exposure prior to pregnancy^35,36^. Moreover, infants of depressed mothers have less synchronous mutual gaze with their mothers than infants of non-depressed mothers^37^. In turn, mutual gaze has been associated with visual attention in the first postnatal year of life^38^. The impact of maternal distress on mother-infant interactions^39^ and maternal sensitivity^40^ have been related to infants’ selective attention^41^ and gaze-following ability^42^.

Though it seems evident that a mother’s mental health, during both antenatal^23,33,34,43–46^ and postpartum^23,37,38,41,45,46^, has a significant impact on offspring’s attention, the underlying mechanisms are still unclear. Previous evidence has shown that the complex and dynamic interactions between multiple biological, psychological and environmental factors contribute to both mothers’ mental health and children’s attention. For example, based on a reciprocal model, children’s attention problems might worsen mothers’ mental health, and in return affect mothers’ mental health that leads to influencing children’s attention^47–49^. Evidence supporting an association between maternal distress and neurological development, more broadly, indicates changes in cortical and subcortical connectivity in human infants^50,51^ and children^52^, and negative impact on neurogenesis and gene expression in neonates of rodents^53^. From biological and environmental perspectives, while TPH1 enzyme mutations in mothers relating to impaired maternal serotonin production increase in a higher risk of inattention in their children^54^, adoption studies demonstrated environmental but genetic factors are associated with children’s ADHD symptoms^55,56^. It has also been reported that increased cortisol levels during the 2nd trimester and increased subjective maternal distress in the 3rd trimester are associated with weaker connectivity of the anterior cingulate cortex of neonates^51^. The anterior cingulate cortex has been linked to infant’s attention^57^, and ADHD in children^58^ and adults^59^. Intriguingly, one previous study investigating infants’ cognitive development at 12 months of age reported that high cognitive performance is linked to lower maternal cortisol levels in the 2nd trimester and higher cortisol levels in the 3rd trimester^60^, suggesting that the link between mother’s cortisol levels and children’s cognitive development is not linear.

Another layer of the complexity comes from the interactions and/or comorbidity between different aspects of maternal psychological distress at different time points as well as whether the symptoms are chronic or not. In particular, adverse childhood experiences that continue to contribute to psychological distress later in life are common. Previous studies demonstrated that the severity of psychological distress is strongly linked to exposure to traumatic experiences earlier in life^61,62^. Studies also suggested that different types of traumatic events, such as interpersonal and non-interpersonal trauma, have different impacts on mental distress and psychiatric symptoms^63,64^. This moderation effect of adverse childhood experiences on maternal psychological distress might result from the alternation of hypothalamic–pituitary–adrenal axis functioning^65^ and increased sensitivity to negative cues^66^, in turn, leads to increase anxiety^66,67^. In addition, evidence shows that individual differences contribute to different trajectories related to life satisfaction and well-being after traumatic events^68^, some individuals develop higher risks in posttraumatic stress disorder, depression, and anxiety^69^. Thus, when studying the relationships between different aspects of maternal mental health and infants’ attention, it is crucial to take maternal adverse childhood experiences into account.

Taken together, compelling evidence has shown that early adverse experiences increase the risks of depression and anxiety later in life^70,71^ and during different perinatal phases^72,73^; and maternal psychological distress significantly hinders the development of attention in childhood^23–26^. However, the overarching effect on focused attention in infancy, a time period when the brain is highly plastic, remains less understood. Besides the challenges of assessing infants’ attention, it is very difficult to disentangle the effects from different aspects of maternal distress (e.g. types and timing), biological, and environmental factors. The analysis of multiple risk factors together is essential due to the high likelihood of comorbidity and high correlations between risk factors. To distinguish possible interactions between different aspects of maternal mental health on infants’ attention will be beneficial for targeted prevention and early intervention. Hence, to better understand the possible underlying mechanisms and examine whether early childhood adverse experiences affect infants’ attention, our longitudinal study narrowed down to access the full path of mothers’ depressive and anxiety symptoms from the 2nd trimester to 6 months postpartum as well as mothers’ childhood traumatic exposure. This time window focused on the in-utero period and the first 6 months postpartum, a period when most infants and mothers share proximal contacts, allowing us to address the maternal-specific factors and to study their associations with infants’ focused attention from 6 to 18 months. When relating mothers’ mental health to infants’ attention, we used a robust focused attention index based on a data-driven method combing fixation data from a wide range of audio-visual tasks. We hypothesize that maternal childhood trauma exposure contributing to maternal distress negatively affects infant’s attention.

## Results

### Multivariate regression analysis

**Figure 1:**
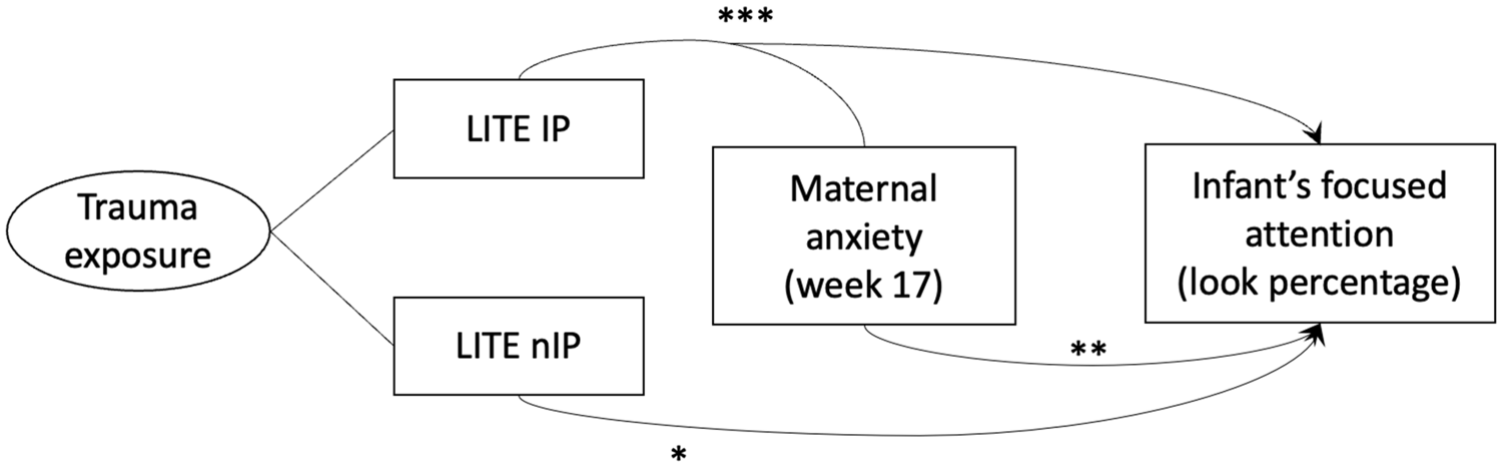
Illustration of the multivariate linear regression after adjusting for the sex of infant, mother’s education level, smoking history, and maternal age at birth. Non-interpersonal traumatic experiences in mother’s childhood and maternal anxiety in early pregnancy had a direct impact on infants’ look percentage in the interaction model. When anxiety at week 17 of pregnancy interacts with interpersonal traumatic exposure in childhood, the negative association with the infants’ look percentage is highly significant. *LITE* Life Incidence of Traumatic Events; *IP* interpersonal events; *nIP* non-interpersonal events.

As seen in Table 1, Model A (*F*(5, 104) = 4.479, *R*^*2*^ = 0.177, *p* < 0.001) includes all significant variables systematically selected from Table 2 as described in the Methods. We observed that higher levels of interpersonal traumatic experience in childhood interact with anxiety during the 2nd trimester and a decrease in infants’ attention (see Model A in Table 1, *b* =−0.038, *p* < 0.001). We also found two main effects. First, when mothers were exposed to higher levels of non-interpersonal trauma in childhood, there was a decrease in infants’ attention to audio-visual stimuli (*b* =−0.029, *p* = 0.011). Second, when mothers reported higher levels of anxiety during the 2nd trimester, infants increased their attention (*b* = 0.055, *p* = 0.003). Unlike the first main effect showing the same direction as in the correlational result (*r* = −0.03, *p* = 0.02), the second main effect is only evident in the presence of the interaction in the model. The second step, Model B (*F*(4, 105) = 5.287, *R*^*2*^ = 0.168, *p* < 0.001) contained only variables that were significant predictors in Model A. All effects remained significant in Model B: the interaction between interpersonal traumatic events and anxiety level during the 2nd trimester (*b* =−0.039, *p* < 0.001), the main effect of non-interpersonal traumatic events (*b* =−0.029, *p* = 0.011), and the main effect of anxiety level during the 2nd trimester (*b* = 0.051, *p* = 0.005). After controlling for infant’s sex, mother’s education, smoking habits, and maternal age at birth, Model C (*F*(8, 99) = 2.888, *R*^*2*^ = 0.189, *p* = 0.006) showed that the interaction between interpersonal traumatic experiences and anxiety during the 2nd trimester (*b* =−0.040, *p* < 0.001), the main effect of non-interpersonal traumatic events (*b* =−0.029, *p* = 0.014), and the anxiety level during pregnancy during 2nd trimester (*b* = 0.052, *p* = 0.006) all remained significant. Figure 1 visualizes the results of Model C.

**Table 1:** The final multivariate linear model with infants’ look percentage as an outcome measure.

Model	Variables	Estimate	SE	Std. Beta	*t* value	*p* value	Model summary
A	(Constant)	0.805	0.022		36.702	< 0.001	F (5, 104) = 4.479, *p* < 0.001, *R*^*2*^ = 0.177
	nIP	−0.029	0.011	−0.235	−2.582	0.011	
	BAI w17	0.055	0.018	0.887	3.024	0.003	
	EPDS w17	−0.008	0.007	−0.131	−1.099	0.274	
	IP	−0.004	0.011	−0.036	−0.389	0.698	
	IP*BAI w17	−0.038	0.011	−0.991	−3.408	< 0.001	
B	(Constant)	0.804	0.022		36.662	< 0.001	F (4, 105) = 5.287, *p* < 0.001, *R*^*2*^ = 0.168
	nIP	−0.029	0.011	−0.237	−2.599	0.011	
	IP	−0.004	0.011	−0.035	−0.378	0.706	
	BAI w17	0.051	0.018	0.832	2.874	0.005	
	IP*BAI w17	−0.039	0.011	−1.023	−3.534	< 0.001	
C	(Constant)	0.820	0.055		14.791	< 0.001	F (8, 99) = 2.888, *p* = 0.006, *R*^*2*^ = 0.189
	nIP	−0.029	0.012	−0.232	−2.495	0.014	
	IP	−0.002	0.012	−0.015	−0.152	0.880	
	BAI w17	0.052	0.018	0.843	2.833	0.006	
	IP*BAI w17	−0.040	0.011	−1.058	−3.572	< 0.001	
	Infant’s sex	0.001	0.012	0.005	0.50	0.960	
	Mother’s education	0.022	0.014	0.164	1.588	0.115	
	Mother’s smoking habit	0.004	0.013	0.028	0.274	0.785	
	Mother’s age at birth	−0.002	0.002	−0.129	−1.300	0.197	

Model A includes all significant variables united from Table 2. Model B uses the backward stepwise method to eliminate variables and improve the model. Model C is the final model after adjusting for infant sex, mother’s education, smoking habit, and the mother’s age birth.

SE, Standardized Error; Std. Beta, Standardized Beta; EPDS, Edinburgh Postnatal Depression Scale; BAI, Beck Anxiety Inventory; w17, pregnancy week 17; w32, pregnancy week 32; pv6, postpartum 6 weeks; pm6, postpartum 6 months; IP, interpersonal events; nIP, non-interpersonal events.

**Table 2:** Four separated multivariable linear regression models for systematically selecting variables for the final model.

Model	Initial included independent variables	Independent variables after backward stepwise elimination	Estimate	Std. Error	Std. Beta	t value	*p* value	Model summary
Non-interpersonal traumatic events and depression	nIP, EPDS w17, EPSD w32, EPDS pw6, EPDS pm6, nIP*EPDS w17, nIP*EPSD w32, nIP*EPDS pw6, nIP*EPDS pm6	(Constant)	0.795	0.018		44.636	< 0.001	*F*(3, 106) = 3.602, *p* = 0.015, corrected *p* = 0.04, *R*^2^ = 0.0925
		nIP	−0.029	0.012	−0.233	−2.507	0.014	
		EPDS w17	−0.012	0.006	−0.199	−2.145	0.034	
		EPDS pm6	0.006	0.006	0.098	1.053	0.295	
Interpersonal traumatic events and depression	IP, EPDS w17, EPSD w32, EPDS pw6, EPDS pm6, IP*EPDS w17, IP*EPSD w32, IP*EPDS pw6, IP*EPDS pm6	(Constant)	0.754	0.006		130.371	< 0.001	*F*(3, 106) = 2.936, *p* = 0.037, corrected *p* = 0.73, *R*^2^ = 0.076
		EPDS w17	0.026	0.019	0.422	1.415	0.160	
		IP*EPDS w17	−0.025	0.012	−0.634	−2.125	0.036	
Non-interpersonal traumatic events and anxiety	nIP, BAI w17, BAI w32, BAI pw6, BAI pm6, nIP*BAI w17, nIP*EPSD w32, nIP*BAI pw6, nIP*BAI pm6	(Constant)	0.794	0.018		44.241	< 0.001	*F*(3, 106) = 2.936, *p* = 0.037, corrected *p* = 0.73, *R*^2^ = 0.077
		nIP	−0.028	0.012	−0.229	−2.445	0.016	
		BAI w17	−0.009	0.006	−0.147	−1.563	0.121	
		BAI pm6	0.006	0.006	0.098	1.046	0.298	
Interpersonal traumatic events and anxiety	IP, BAI w17, BAI w32, BAI pw6, BAI pm6, IP*BAI w17, IP*EPSD w32, IP*BAI pw6, IP*BAI pm6	(Constant)	0.756	0.006		132.995	< 0.001	*F*(4, 105) = 3.906, *p* = 0.005, corrected *p* = 0.02, *R*^2^ = 0.130
		BAI w17	0.051	0.018	0.829	2.812	0.006	
		BAI pw6	−0.008	0.005	−0.136	−1.496	0.181	
		IP*BAI w17	−0.039	0.011	−1.001	−3.396	0.001	

Look percentage is the common dependent variable in all four models. Significant variables of each model are included in the united model. Corrected *p* value is calculated based on the Holm-Sidak method.

Std. Error, Standardized Error; Std. Beta, Standardized Beta; EPDS, Edinburgh Postnatal Depression Scale; BAI, Beck Anxiety Inventory; w17, antenatal 17 weeks; w32, antenatal 32 weeks; pv6, postpartum 6 weeks; pm6, postpartum 6 months; IP, interpersonal traumatic events; nIP, non-interpersonal traumatic events.

### Moderation analysis

**Figure 2:**
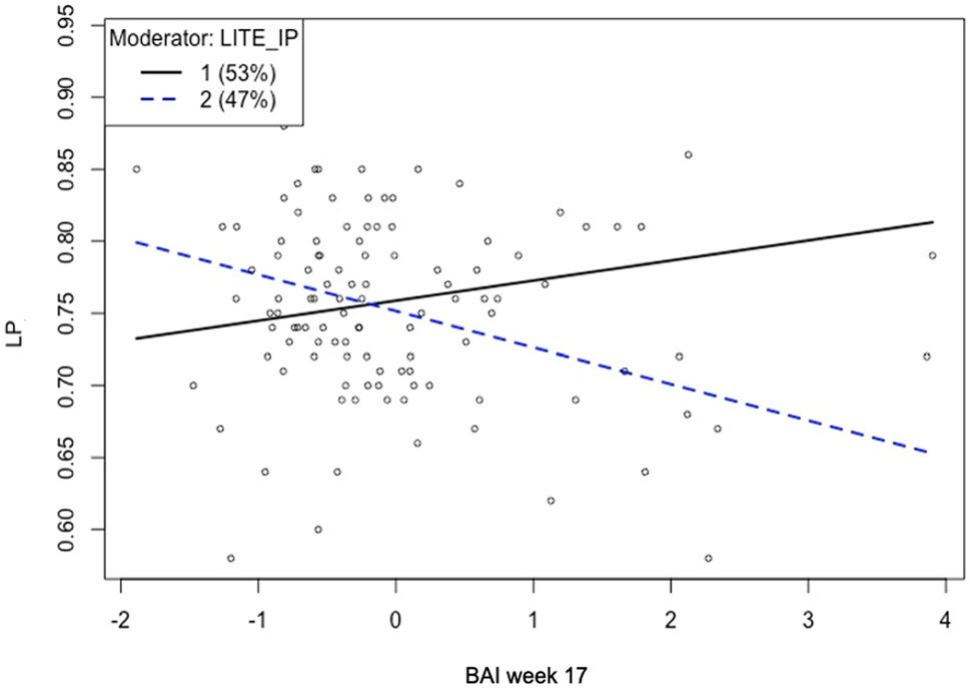
The relationship between maternal anxiety at antenatal 17 weeks (Beck Anxiety Inventory, BAI week 17) and infant’s focused attention (look percentage, LP), is moderated by the level of interpersonal traumatic events (IP) in mother’s childhood measured by Life Incidence of Traumatic Events (LITE). Level 1 (solid line) represents mothers who exposed to less trauma in childhood compared to those at level 2 (dotted line).

Following the results described above, exposure to interpersonal traumatic events in childhood was examined as a moderator of the relationship between the anxiety level during the 2nd trimester and the infants’ look percentage after adjusting for infant sex and mother’s education. Figure 2 displays the slopes for the anxiety level during antenatal 17 weeks and the levels of the exposure to interpersonal traumatic events predicting infants’ attention. As indicated by the change in the direction, the effect is moderated by interpersonal traumatic events (*F*(5, 103) = 2.916, *R*^*2*^ = 0.124, *p* = 0.017). In other words, the strength of the association between maternal anxiety and infant’s focused attention is stronger amongst those with higher maternal exposure to childhood traumatic events compared to those with lower exposure.

## Discussion

The primary goal of the current study was to investigate whether maternal distress affects the development of focused attention in infancy. We found that exposure to non-interpersonal and interpersonal traumatic experiences in childhood is associated with infants’ focused attention. Moreover, childhood interpersonal trauma experience moderates the maternal anxiety level during the 2nd trimester showing the negative impact on the development of focused attention in infancy. Our results expand our understanding of the impact of maternal adverse childhood experiences on infants’ outcomes, and the possible mechanisms driven by maternal anxiety. From the standpoint of prevention, our findings underscore the importance of early screening and intervention for mental health issues to support mothers and infants and prevent long-term consequences, even before the pregnancy starts.

Based on our findings, one critical aspect shows that adverse childhood experiences, in particular the interpersonal traumatic events, might foster the negative impact of maternal anxiety on infants’ focused attention. Literature suggests that early traumatic experiences contribute to the change of limbic reactivity^74^ and fronto-limbic circuit^75^ which are related to dysfunction of emotional regulation^76^. There is also evidence that compared to the exposure to non-interpersonal trauma experience, the exposure to interpersonal trauma is associated with the higher levels of sensitivity to punishment^77^, guilt, and shame^64^. Those multiple factors may lead to a long-term cumulative effect of maladaptation and anxiety^78^, which in turn, affect infants’ outcomes. A recently proposed model in line with the fetal programming framework suggests that fetal life represents a particularly sensitive period when the effects of maternal adverse childhood experiences could be transmitted through psychological, biological, biophysical, and behavioral sequelae^79^. In fact, the fetus’s growth of grey matter accounts for the total cerebral growth significantly in the second half of pregnancy^80^. A recent study reported that maternal adverse childhood experiences may lower newborn’s intracranial volume and change the trajectory of cortical gray matter growth, suggesting there are structural neurodevelopmental consequences in in-utero resulting from maternal childhood trauma^81^.

In our longitudinal data across pregnancy to early infancy, we found a particular vulnerability in the 2nd trimester. One most plausible explanation is that the fetal brain is vulnerable to the in-utero environment due to the critical period of neurogenesis. Especially, the development of neuron connectivity, limbic system, hypothalamic–pituitary–adrenal axis, and prefrontal cortex may be disturbed by antennal anxiety and stress (see review by Van den Bergh et al.)^82^. Compared to the 1st and 3rd trimesters, exposure to ethanol in the 2nd trimester has been reported to cause a great neuronal loss in rodents^83^, attenuated cerebral blood flow^84^, and long-lasting alternations in synaptic plasticity^85^ in the human fetus. In children, a previous study also reported that maternal anxiety during the 2nd trimester, but not later during pregnancy, is associated with gray matter reduction in several brain areas in children (6–9 years old)^86^, including the prefrontal lobe, which is a crucial area in cognitive development^87^ and controls attention^88^.

Another possible explanation is associated with elevated cortisol levels in mothers during the 2nd trimester. Previous studies reported that increased cortisol levels during the 2nd trimester are associated with weaker neural connectivity in the anterior cingulate cortex of neonates^51^ and lower cognitive function at 12 months of age^60^. However, increased cortical levels during the 3rd trimester are beneficial for the fetus’s brain maturation and cognitive functions at 12 months of age and later in childhood^60,89^ suggesting that the maternal cortisol levels affect offspring’s cognitive development differently at different time points. More research clarifying the associations might help understand fetal and infant’s developmental changes related to the amount and the timing of cortisol exposure.

Interestingly, maternal depression showed no association with infants’ attention. However, previous literature has shown that infants of depressed mothers have a less synchronous gaze in the mother-infant interaction^37,90^ that may affect the development of attention^38^. Similar to two well-controlled studies investigating cognitive development, maternal depression during pregnancy and infancy did not affect cognitive development at the age of 3 years^91^ and 18 months^92^, respectively. In the context of the current study, there are several plausible reasons for this finding. First, the association between maternal depression and infants’ attention may not exist. However, using the same dataset investigating gaze following, infants of mothers with lower levels of postpartum depression presented better skills in synchronizing visual attention with others based on their gaze direction^42^. Though mutual gaze interaction can predict attention in infancy^38^, our data and Astor et al.’s study^42^ show that there may be more than one pathway of mother-infant interaction that influences the development of attention. Second, it is possible that the impact of maternal depression on infants’ attention is cumulative and becomes significant only in childhood^26^. Third, as maternal depression is complex and heterogeneous in nature^93–95^, our four time points may not reflect the complexity and heterogeneity of associations across mothers and infants. Lastly, because of the rigorous nature of the BASIC study, among mothers with depressive symptoms, a higher proportion of those with high functioning/cognitive skills (of which the children might also have good attention) could have filled out the questionnaires, introducing a possible selection bias.

Keeping these alternatives in mind, we cautiously propose another reason. Given the high comorbidity of depression and anxiety in our data (Table 4) and the literature^96,97^, we propose that anxiety may be the driving force behind peripartum depression. For example, when we examined depressive and anxiety symptoms separately (Table 2), they showed a unique effect during the 2nd trimester. When we further combined all dimensions and examined the effect while simultaneously controlling others, anxiety dominated the effect. To the best of our knowledge, maternal depression and anxiety are rarely combined and related to child development, meaning that the importance of maternal anxiety may have been interpreted as an effect of depression in prior work. However, the complexity and dynamics between traumatic experiences, depression, and anxiety and how the dynamics change over time are beyond the scope of the current study. Future studies are needed to help us understand how maternal mental health affects infants’ attention. Most importantly, it will provide us with more knowledge on promoting maternal mental health and infant development.

Finally, and especially due to our limited sample size, our results must be interpreted in light of some limitations. For the focused attention measure, we applied a data-driven method to explore and establish the focused attention index (look percentage). Theoretically, this index mimics measuring the duration of time an infant spends on targeted tasks. Our method included a great amount of fixation data from several audio-visual tasks and examined the looking behavior at the micro-level. We excluded very few trials where no fixations were present, ensuring that all trials where infants provide valid fixation data are included while avoiding making assumptions about the reason why some trials lack data altogether (e.g. lack of data = poor attention). Combining fixation data across multiple tasks increases the resolution of individual differences though we did not examine the success rate of each trial. Future research might want to investigate the associations between a global focused attention index, such as our look percentage, and looking patterns, success rate, and others that are more task-specific to understand different aspects of the cognitive operation. Another limitation with regard to the attention measure is that we used a composite score. In our results, high internal consistency of the development of focused attention from 6, 10 to 18 months motivated us to create the focused attention as a single construct. Thus, a composite score was calculated. Though this provided us with a straightforward way to explain our results, we might lose some information related to developmental changes. Future longitudinal studies might be interested in emphasizing the developmental trajectories within and between different constructs of attention and investigating their relationships to maternal mental health.

With regard to maternal measures, overall, we focused on the period between pregnancy and the first 6 months postpartum to possibly eliminate partial mother–child reciprocal influences often observed in studies in childhood. Admittedly, in the first year postpartum, there is also evidence demonstrating, for example, that infants’ temperament^98^, has a reciprocal influence on both mother’s mental health and infant’s development. Yet, evidence related to infants’ attention is still scarce.

Another limitation is that we could not focus on clinically severe cases due to the relatively small number of severely depressed mothers. To deal with the relatively small sample size and the significant collinearity between depression and anxiety, we calculated factor scores for depression and anxiety separately at four different time points. This may prevent interactions at different stages and different levels to impact the results in unforeseeable ways.

In addition, we used a dichotomous distinction to separate groups of mothers with low- vs high-traumatic exposure. There are advantages and disadvantages to this strategy. We are aware that using dichotomic variables reduces variability in the data. At the same time, our data showed a low rate of different frequencies, dichotomization made it simpler to study and interpret interaction effects. Alternatively, future studies might use the raw scores or convert them to other continuous values.

Moreover, our sample is limited to a homogenous population in Uppsala (Sweden), with more than half of participating mothers having education levels of university or higher. Furthermore, we did not control for the possible influence of partners’ mental health on mothers’ well-being and infants’ attention. As our results indicate the important influence of interpersonal traumatic experiences, future studies should consider this interpersonal aspect and its dynamics with regard to mothers’ well-being.

Our findings add to the growing body of research, suggesting that prevention and intervention should start before pregnancy for both mothers and infants. Lastly, the findings describe a previously undocumented connection between maternal early trauma, anxiety, and the development of focused attention in infants. Treating pregnant women’s anxiety, especially if she has experienced traumatic events in the past, may not only improve the lives of mothers but also support the positive development of their children from infancy onwards.

## Methods

### Participants

The final data included 118 mother-infant dyads from the BASICchild cohort as part of a longitudinal study (the BASIC Child Project)^99^ of a subsample of the population-based BASIC study "Biology, Affect, Stress, Imaging, and Cognition (BASIC)"^100^ collected from 2014 to 2018. Characteristics of the mother-infant dyads are shown in Table 3. Only healthy pregnant women > 18 years old who received a routine examination at Uppsala University Hospital were invited to participate in the projects. Mothers who consented to participate were invited to fill out a series of questionnaires online at 17 and 32 gestational weeks, and postpartum at 6 weeks, 6 months, and 12 months. Mothers and infants who took part in the BASIC Child Project visited the Uppsala Child and Baby Lab when the infants were aged 6 (*n* = 118; mean = 185 days, SD = 7.5 days, 59 boys), 10 (*n* = 110; mean = 302 days, SD = 9.2 days, 53 boys), and 18 months (*n* = 104; mean = 544 days, SD = 12.1 days, 53 boys). All infants were reported healthy. Sixty-five percent of the mothers held a university degree. All procedures in the study were conducted in accordance with the 1964 Declaration of Helsinki ethical standards and approved by the Regional Ethical Review Board in Uppsala, Sweden (EPN). Mothers who agreed to participate in the online surveys returned their written informed consent prior to the study. For participating infants, all legal guardians provided written informed consent during each visit prior to the experiment. Participants received a gift voucher worth approximately 30 euros after each visit to the lab.

**Table 3:** Demographic characteristics of 118 mother-infant dyads.

Characteristic	Mother-infant dyad (*n* = 118)
Maternal age, years	30.54 (3.92)
Country of origin	
Scandinavian	93.1%
Other	6.9%
Maternal education	
University or higher	65.0%
Other	35.0%
Cohabitating with the second caregiver	99.2%
With smoking history	36.4%
Employment	
Full-time	61.2%
Part-time	18.1%
Student	9.5%
Sick leave	4.3%
Unemployed	6.9%
Length of gestation, days	280 (8.09)
Infant sex, female	59%
Infant birth weight, g	3,664 (481)
Infant’s Apgar score at 5 min	
7	0.9%
8	2.6%
9	6.0%
10	90.6%

Data are given as the proportion of dyads or mean (SD).

### Measures of maternal distress

Symptoms of depression were measured using the Swedish version of the Edinburgh Postnatal Depression Scale (EPDS)^101,102^. The EPDS includes 10 questions scored from 0 to 3. Thus, the total score ranges from 0 to 30, with higher scores indicating more severe symptoms. The reliability and validity of the EPDS have been shown to be adequate^103,104^. Symptoms of anxiety were measured using the Beck Anxiety Inventory (BAI)^105^. The scale consists of 21 items, with participants indicating the extent to which they were bothered by each item. The total score for each item ranges from 0 to 63, with higher scores indicating more server symptoms^106^. A high level of internal consistency and a good test–retest correlation have been reported^105^. Mothers in the study completed the online version of both EPDS and BAI at 17 and 32 weeks of pregnancy and 6 weeks and 6 months of the first postnatal year. Childhood traumatic exposure was measured using the Swedish version of the Life Incidence of Traumatic Events (LITE)^107,108^. The LITE is a self-reported checklist that consists of 15 fixed items and one optional item. Each item enquires whether the event has occurred, how many times, the age of the first occurrence, and how inconvenient it remains now. The first eight items ask whether different types of non-interpersonal traumatic events (nIP) have occurred, whereas the remaining items ask whether the seven types of events regarding interpersonal traumatic events (IP) occurred. Interpersonal events are defined as events dependent on a conscious act of another human being, such as physical harm, divorce, or separation of parents, etc. Non-interpersonal events include natural disasters, accidents, or illness of others, etc. The sums of occurrences of nIP and IP were used as two variables in the analysis. Acceptable test–retest reliability and validity have been reported^109^. Mothers in the current study were invited to complete the LITE online during postpartum 12 months.

### Measure of infants’ focused attention

Infants’ focused attention was measured by the look percentage (defined as the total fixation duration of the stimuli divided by the total duration of all tasks within the same age group) across a variety of free-looking tasks at the age of 6, 10, and 18 months (see Supplementary Table S1). All tasks were presented as dynamic audio-visual stimuli. During each visit, infants were invited to watch a serial of videos that were divided into 3 to 4 blocks. Each block lasted between 5 to 7 min. Conceptually, focused attention is the ability to focus and spend a period of time on targeted tasks^2,8,110^. In the current study, we applied a data-driven method and determined the measure, look percentage, that mimics the theoretical concept to evaluate focused attention. Overall, there were ca. 0.51 million fixations included in the final analysis (further information about the missing trials across tasks at different age points please see Supplementary Table S2). There are a few reasons for our choice to combine a theoretical-based and a data-driven method. First, data-driven methods are regularly applied in the field. They provide the opportunity to explore data while relaxing theory-driven constraints with more freedom and allowing new knowledge to merge^111^. We believe that the field can benefit from examining looking data from a different perspective. Second, previous studies have demonstrated that individual looking or fixation duration is stable and consistent^112,113^ across stimuli types in early development^113,114^. Third, when we preprocessed the mean and variance of fixation duration across tasks with the same age group, we observed consistency in the results (see Supplementary Figure S1). Taken together, we aggregated all fixation data from different tasks within the same age point for further analysis. Outliers were removed using a z-score. The age-appropriate tasks are listed in Supplementary Table S1. A series of videos depicting the stimuli presented to participants can be viewed on Databary as following https://nyu.databrary.org/volume/828.

In this study, the mean look percentage at 6, 10, and 18 months was 73.63% (SD = 9.84), 73.47% (SD = 9.36), and 79.24% (SD = 6.86), respectively. The Pearson’s correlation coefficients (Table 4) of attention, look percentage, between different ages were 0.33 (6–10 months, *n* = 110, *p* < 0.001), 0.21 (6–18 months, *n* = 103, *p* = 0.04), and 0.31 (10–18 months, *n* = 100, *p* < 0.01), suggesting the stability and internal consistency of attention during the course of development. In the current study, the composite score of look percentage was calculated by averaging each participant’s look percentage measured at three time points and used as the dependent variable. There are two reasons that a composite score is used. First, from the correlational results, look percentages between different age points are very consistent, suggesting the individual difference is stable across three time points and it is reasonable to create a single construct. Second, to answer our research question, we used the focused attention measure to relate to 10 maternal variables. Reducing the number of variables is helpful to reduce the complexity of regression analysis and to better interpret the results. All tasks were recorded using an eye-tracker with a sampling rate of 60 Hz following a 5-point calibration (Tobii TX300, Tobii Technology AB, Sweden).

**Table 4:** Pearson’s zero order correlations between all variables using raw scores.

	Timing of measure	1	2	3	4	5	6	7	8	9	10	11	12	13	14
1. LP (6 months)	Postpartum	–													
2. LP (10 months)	Postpartum	0.33***	–												
3. LP (18 months)	Postpartum	0.21*	0.31**	–											
4. LP composite	Postpartum	0.76***	0.79***	0.62***	–										
5. EPDS w17	Antenatal	–	–	–	–	–									
6. EPDS w32	Antenatal	–	–	–	–	0.75***	–								
7. EPDS pw6	Postpartum	–	–	–	–	0.47*****	0.58***	–							
8. EPDS pm6	Postpartum	–	–	–	–	0.54***	0.57****	0.63***	–						
9. BAI w17	Antenatal	–	–	–	–	0.74***	0.60***	0.40***	0.37***	–					
10. BAI w32	Antenatal	–	–	–	–	0.57***	0.68***	0.41***	0.41***	0.76***	–				
11. BAI pw6	Postpartum	–	–	–	–	0.43***	0.49***	0.62***	0.54***	0.53***	0.54***	–			
12. BAI pm6	Postpartum	–	–	–	–	0.37***	0.49***	0.40***	0.59***	0.52***	0.53***	0.66***	–		
13. LITE IP	Postpartum	–	–	–	–	0.22*	0.24*	0.25**	0.25*	0.21*	0.19*	0.34***	–	–	
14. LITE nIP	Postpartum	–	−0.17^**+**^	−0.35***	−0.26**	–	–	–	–	–	–	–	–	0.34***	–
Skewness	–	−0.87	−0.52	−0.85	−0.47	0.95	0.97	0.79	0.95	1.47	1.06	1.45	1.57	0.95	0.97
Kurtosis	–	1–52	−0.16	1.09	−0.03	0.68	1.33	0.01	0.88	2.46	1.12	1.90	2.40	0.68	1.33
VIF 1	–	NA	NA	NA	NA	5.24	3.95	2.50	2.86	4.23	2.83	2.78	2.98	1.24	1.23
VIF 2	–	NA	NA	NA	NA	2.05	1.43	1.40	1.54	2.01	1.47	1.50	1.50	1.13	1.07
MSA	–	NA	NA	NA	NA	0.70	0.71	0.76	0.77	0.72	0.72	0.77	0.77	0.42	0.43

^**+**^ *p* < 0.1, * *p* < 0.05, ** *p* < 0.01, *** *p* < 0 .001 with Benjamini–Hochberg correction. Abbreviations: LP, look percentage; LP composite, mean look percentage of three age points; EPDS, Edinburgh Postnatal Depression Scale; BAI, Beck Anxiety Inventory; LITE, Lifetime Incidence of Traumatic Events; VIF, variance inflation factor (using LP as an outcome, other 10 variables as predictors; VIF 1 is calculated all with raw scores; VIF 2 is calculated with factor scores of EPDS and BAI and composite scores of LITE); MSA, measure of sampling adequacy according to Kaiser–Meyer–Olkin test; w17, pregnancy week 17; w32, pregnancy week 32; pv6, postpartum 6 weeks; pm6, postpartum 6 months; IP, interpersonal events; nIP, non-interpersonal events; NA: not applicable, as LP 6, 10, and 18 months were used as dependent variables.

### Statistical analysis

#### Maternal psychological distress

We used multivariate linear regression models and a moderator analysis to examine the association between multiple predictors across different time points and the outcome measure. To assess the reliability of the maternal scale instruments, we calculated the internal consistency coefficient, Cronbach’s alpha for each tool: EPDS, 0.87, good; BAI, 0.81, good; and LITE, 0.9, excellent. Before adjusting their scores, the zero-order Pearson correlations (with Benjamini–Hochberg correction), skewness, and kurtosis of all variables were calculated (Table 4). The variance inflation factor (VIF) was calculated based on the assumption that infants’ look percentage is predicted by 10 variables from the EPDS (4 time points), BAI (4 time points), and LITE (1 time point). For the10 maternal variables, we performed a test of Missing Completely at Random for multivariate data with missing value^115^. Given the *p*-value for the chi-squared statistic was 0.82, we can conclude that maternal variables are missing completely at random. As seen in Table 4, raw scores for anxiety symptoms during antenatal 17 weeks and postpartum 6 weeks are not in the acceptable range of the kurtosis index. The raw scores of the EPDS, BAI, and LITE did not reach the range of approximate symmetric distribution (kurtosis index acceptable range, −2 to + 2; skewness index acceptable range -0.5 to + 0.5)^116^. In addition, the literature has shown that comorbidity of depression and anxiety is common^96,97^, so we expected to detect potential multicollinearity from the raw data. As seen in Table 4, the raw scores of the EPDS and the BAI during antenatal 17 weeks fit the strict criteria for multicollinearity (VIF1 > 4) with other variables^117,118^. Considering the non-normal distribution and multicollinearity of the EPDS and BAI, the Kaiser–Meyer–Olkin test was used to examine the sampling adequacy (MSA) and transformed all raw scores from four time points into factor scores (MSA > 0.65)^119^. The percentage of missing values in EPDS and BAI at 4 different time points are–0.8, 0, 8, 12, and 8, 3, 5, 13.5–, respectively. Missing values were imputed using predictive mean matching^120^. Individual factor scores of the EPDS and BAI at four time points were calculated using the imputed values. The LITE raw scores, including IP and nIP, were the frequency of the occurrences. To be consistent in the analysis using the comparable values that can represent different levels, they were transformed into dichotomic variables based on the median of the raw scores to interpret the interaction. This choice was made due to (1) the asymmetrical distribution of the raw scores (see Table 4, the value of skewness of nIP and IP); (2) the infrequent occurrence of extremely high numbers; (3) low rate of different frequencies; and (4) the lack of a standardized scoring system to distinguish clinically significant levels. To examine differences in high versus low levels of exposure, a dichotomic categorization splitting based on median permitted the comparison between the subgroup never or rarely exposed to trauma and the subgroup that appeared to be frequently exposed to trauma^121,122^. There were 8 data points missing in LITE. Unlike EPDS and BAI covering multiple time points and considering the unknown mechanisms of how trauma is related to other factors, deleting missing data was considered not to over-interpret the data. More details are presented in Supplementary Table S3. The outcome measure was infants’ look percentage composite.

#### Variable elimination and model fitting

Initially, there was a theoretical selection of 10 predictors included in the current data set that evaluated trauma exposure (one time point of previous IP and nIP), depressive symptoms (four time points), and anxiety symptoms (four time points) in the main analysis to predict infants’ look percentage. No other variables except those listed here have been evaluated as part of the analysis. In step 1, considering that maternal trauma exposure prior to pregnancy (both IP and nIP) may interact with depression or anxiety, we separated variables into four groups as listed in Table 2 and analyzed four linear regression models independently. Applying a backward stepwise method, the number of variables in each model was reduced (3rd column, Table 2). In step 2, we performed Holm-Sidak correction to adjust the *p* values of all models^123,124^. Based on the statistical selection shown in Table 2, we combined the significant variables and 2-degree interaction from two significant models to assess how they jointly predict infants’ focused attention (measured by look percentage; see Model A, Table 1). Based on Model A, we selected significant variables for Model B (see Table 1). In the third step, we added the sex of infants^125,126^, mothers’ smoking habits^127^, education^128^, and the maternal age at birth^129^ to the analysis (Model C, Table 1). All tests were two-sided tests with *p* < 0.05 considered significant. All statistical analyses were performed using R 4.0.3^130^.

## Supplementary Information

Supplementary Information.
