# Supplementary material for: Maternal childhood trauma and perinatal distress are related to infants’ focused attention from 6 to 18 months
Source: Sci Rep. 2021 Dec 17;11:24190. doi: 10.1038/s41598-021-03568-2 (PMC8683435; doi:10.1038/s41598-021-03568-2)
Supplement: Supplementary file 1 — Supplementary Information. [file 41598_2021_3568_MOESM1_ESM.docx]

Title: Maternal childhood trauma and perinatal distress are related to infants’ focused attention from 6 to 18 months

**Supplementary Information**

Supplementary Table S1. The audio-visual tasks included in assessing look percentage

| **Task** | **Description** | **Test age *** |
| --- | --- | --- |
| Give-Me | *Give-Me gesture interactions* were used to access action evaluation ^1,2^. A 40-second-context for a give-me gesture followed by appropriate or inappropriate giving was repeated 3 times (26 s in total). Four appropriate and four inappropriate trials were presented. | 6, 10 |
| Change detection task | *Change detection task* modified based on Libertus and Brannon’s study was used to access the ability to discriminate between numericities ^3^. Two image streams simultaneously on both sides of a screen were presented to infants. Images alternated between different numbers of dots with three ratios (1:4, 1:2; 2:3). Each trial lasted for 10 s. | 6, 10, 18 |
| Multimodal events | *Multimodal events* were used to evaluate the ability of associative learning (multimodal events that were binding to locations) ^4^. Infants were shown short video clips that a particular sound was binding to a particular location of a stimulus. | 6, 10 |
| Biological motion | *Biological motion* was used to investigate whether infants’ perception of biological motion ^5^. There were two identical animated human-like stimuli presented side-by-side on the screen. One was upright and the other was reversed. They showed the same movements but in a reversed mirror direction. There was no auditory stimulation involved. | 6, 10 |
| Coherent motion task | *Coherent motion task* was inspired by previous studies and it was to measure infants’ ability to discriminate between two coherent or random movements ^6,7^. Two groups of moving dots were presented on two sides of the screen. One contained dots that all moved in random directions. | 6, 10 |
| Gaze following task | *Gaze following task* was used to examine the degree to which infants follow another person’s gaze ^8,9^. | 6, 10 |
| Pupillary light response | *Pupillary light response* was used to measure the constriction of the pupil diameter in response to a flash of light. | 6, 10, 18 |
| Small forms discrimination task | *Small forms discrimination task inspired by previous studies* was used to investigate infants’ perception and sensitivity of four geometrical forms ^10,11^. In the task, infants were presented with an array of four small forms each containing two connected lines that formed an angle. Each array included three forms that were identical and one form that deviated. | 6, 10 |
| Face perception | *Face perception* was used to access whether infants can perceive emotional expressions in faces. Happy, fearful, and neutral facial expressions of three young women were presented to infants at 6 months. Additional two emotions, sad, and scared expressions were presented to infants at 10 and 18 months. All visual stimuli in this task were from the FACES-database ^12^. | 6, 10, 18 |
| Visual sequence task | Visual sequence task was used to examine if infants can learn the pattern the stimuli were presented ^13^. | 10, 18 |
| Reaching | *Reaching task was used* to access how infants shift their gaze toward a reaching action ^14^. | 18 |

Supplementary Table S2. The number of trials and missing trials in each task by age

| **Age (month)** | **Task** | **Nr. of trials** | **Nr. of average missing trials** |
| --- | --- | --- | --- |
| 6 | Action evaluation rational | 6 | 0.20 |
|  | Action evaluation irrational | 6 | 0.15 |
|  | Approximate number system | 6 | 0.11 |
|  | Face perception (3 emotions) | 12 | 0.31 |
|  | Biological motion | 4 | 0.10 |
|  | Gaze following | 6 | 0.19 |
|  | Small forms discrimination | 8 | 0.23 |
|  | Pupillary light response | 8 | 0.00 |
|  | Action prediction | 2 | 0.00 |
|  | Coherent motion task | 4 | 0.13 |
|  | Associative learning | 2 | 0.01 |
| 10 | Action evaluation rational | 6 | 0.33 |
|  | Actional evaluation irrational | 6 | 0.25 |
|  | Approximate number system | 6 | 0.15 |
|  | Biological motion | 4 | 0.15 |
|  | Gaze following | 6 | 0.25 |
|  | Small forms discrimination | 8 | 0.65 |
|  | Pupillary light response | 8 | 0.00 |
|  | Action prediction | 2 | 0.07 |
|  | Coherent motion task | 4 | 0.30 |
|  | Associative learning | 2 | 0.02 |
|  | Gravity | 8 | 0.37 |
|  | Face perception (4 emotions) | 24 | 2.75 |
|  | Visual sequence task | 15 | 0.01 |
| 18 | Reaching | 8 | 0.13 |
|  | Face perception (4 emotions) | 24 | 2.24 |
|  | Approximate number system | 6 | 2.23 |
|  | Visual sequence task | 15 | 0.02 |

Supplementary Table S3. Raw scores and dichotomic scores of interpersonal and non-interpersonal traumatic events

| **Level** | **IP**  **dichotomic score** | **nIP**  **dichotomic score** | **IP**  **raw score^+^** | **nIP**  **raw score^+^** |
| --- | --- | --- | --- | --- |
|  | Counts (% of total) | | | |
| 0 |  |  | 32 (29.1) | 15 (13.6) |
| 1* | 59 (53.6) | 59 (53.6) | 27 (24.5) | 23 (20.9) |
| 2* | 51 (46.4) | 51 (46.6) | 24 (21.8) | 21 (19.1) |
| 3 |  |  | 14 (12.7) | 22 (20.0) |
| 4 |  |  | 10 (9.1) | 20 (18.2) |
| 5 |  |  | 2 (1.8) | 8 (7.3) |
| 6 |  |  | 1 (0.9) | 0 (0) |
| 7 |  |  | 0 (0) | 1 (0.9) |
| N | 110 | 110 | 110 | 110 |
| Missing | 8 | 8 | 8 | 8 |
| Mean | 1.46 | 1.46 | 1.57 | 2.35 |
| Median | 1.00 | 1.00 | 1.00 | 2.00 |
| SD | 0.50 | 0.50 | 1.43 | 1.57 |
| Minimum | 1 | 1 | 0 | 0 |
| Maximum | 2 | 2 | 6 | 7 |
| **Level 1 and 2 as dichotomic scores are based on median split. Level 1 indicates lower level of exposure compared to level 2.*  *+Raw scores are rated based on the frequency of occurred events.*  *Abbreviation: IP, interpersonal traumatic events; nIP, non-interpersonal traumatic events.* | | | | |

Supplementary Figure S1. Illustration of the distributions of mean (left column) and variance (right column) of fixation durations at three different age points across different tasks.


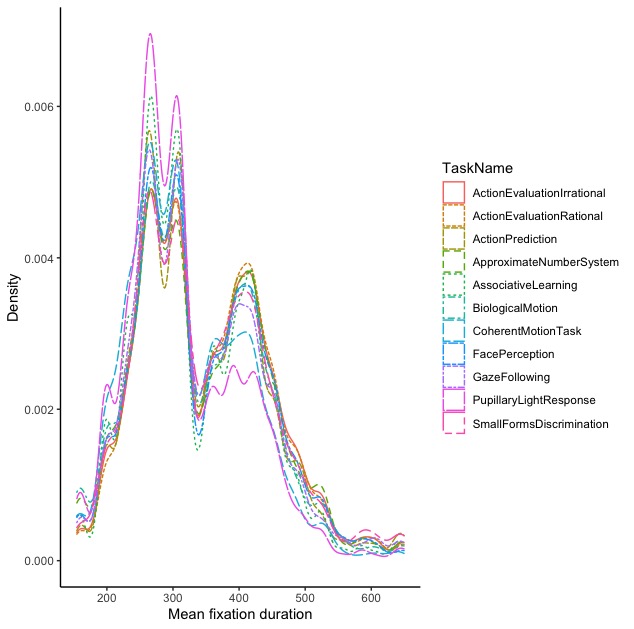

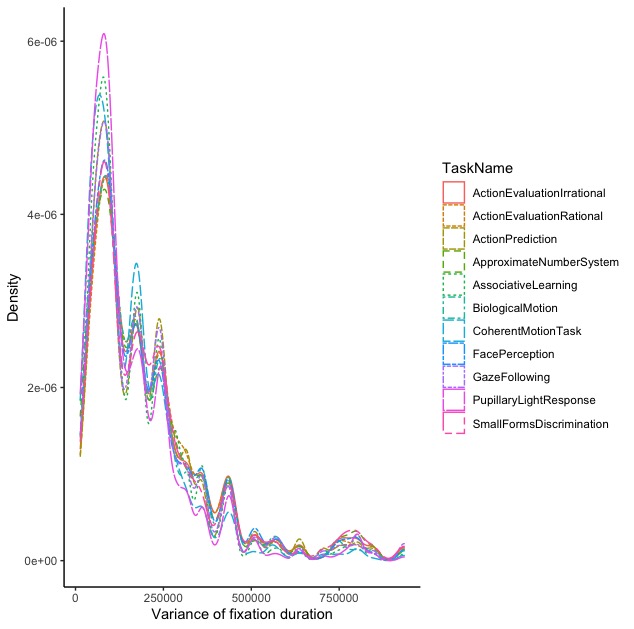

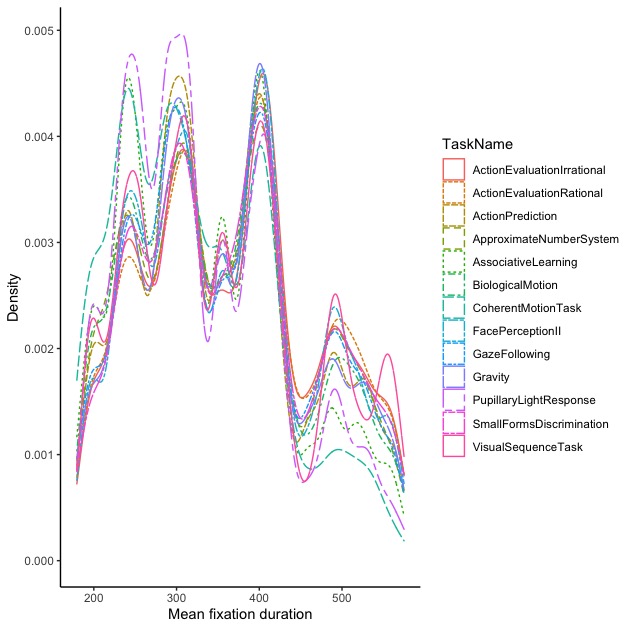

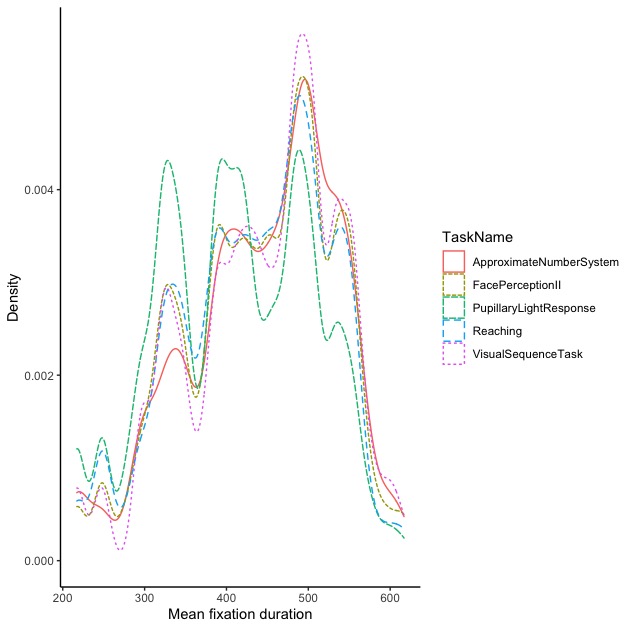

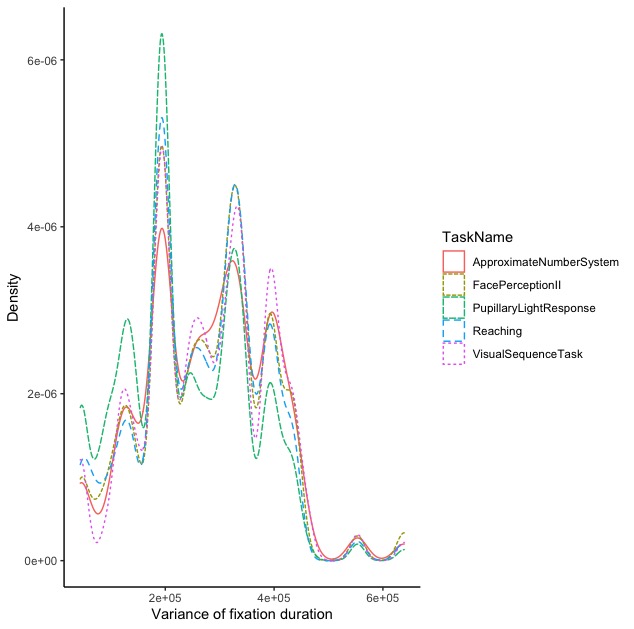

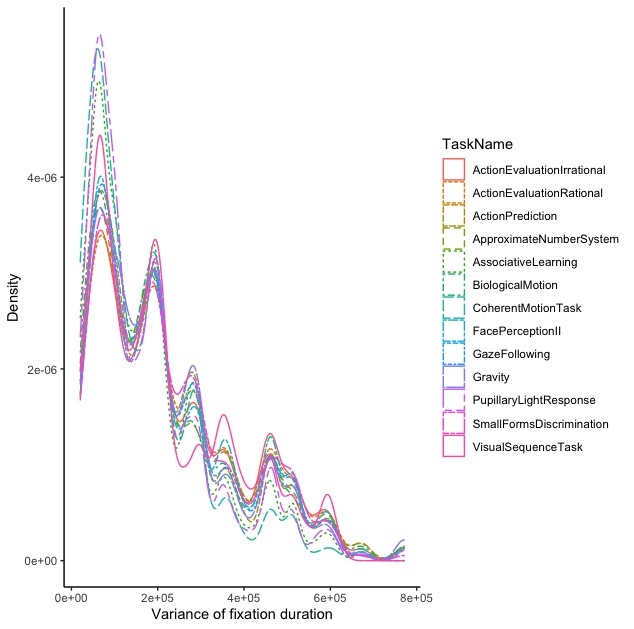


6 months

10 months

18 months

Mean

Variance

References

1 Juvrud, J. *et al.* Longitudinal continuity in understanding and production of giving‐related behavior from infancy to childhood. *Child development* **90**, e182-e191 (2019).

2 Gredebäck, G. & Melinder, A. Infants’ understanding of everyday social interactions: A dual process account. *Cognition* **114**, 197-206 (2010).

3 Libertus, M. E. & Brannon, E. M. Stable individual differences in number discrimination in infancy. *Developmental science* **13**, 900-906 (2010).

4 Richardson, D. C. & Kirkham, N. Z. Multimodal events and moving locations: Eye movements of adults and 6-month-olds reveal dynamic spatial indexing. *Journal of Experimental Psychology: General* **133**, 46 (2004).

5 Falck‐Ytter, T. *et al.* Reduced orienting to audiovisual synchrony in infancy predicts autism diagnosis at 3 years of age. *Journal of Child Psychology and Psychiatry* **59**, 872-880 (2018).

6 Wattam-Bell, J. Coherence thresholds for discrimination of motion direction in infants. *Vision research* **34**, 877-883 (1994).

7 Wattam-Bell, J. *et al.* Reorganization of global form and motion processing during human visual development. *Current Biology* **20**, 411-415 (2010).

8 Gredebäck, G., Astor, K. & Fawcett, C. Gaze following is not dependent on ostensive cues: a critical test of natural pedagogy. *Child Development* **89**, 2091-2098 (2018).

9 Szufnarowska, J., Rohlfing, K. J., Fawcett, C. & Gredebäck, G. Is ostension any more than attention? *Scientific Reports* **4**, 5304 (2014).

10 Dillon, M. R., Huang, Y. & Spelke, E. S. Core foundations of abstract geometry. *Proceedings of the National Academy of Sciences* **110**, 14191-14195 (2013).

11 Izard, V. & Spelke, E. S. Development of sensitivity to geometry in visual forms. *Human Evolution* **23**, 213 (2009).

12 Ebner, N. C., Riediger, M. & Lindenberger, U. FACES—A database of facial expressions in young, middle-aged, and older women and men: Development and validation. *Behavior research methods* **42**, 351-362 (2010).

13 Sheese, B. E., Rothbart, M. K., Posner, M. I., White, L. K. & Fraundorf, S. H. Executive attention and self-regulation in infancy. *Infant Behavior and Development* **31**, 501-510 (2008).

14 Henrichs, I., Elsner, C., Elsner, B., Wilkinson, N. & Gredebäck, G. Goal certainty modulates infants’ goal-directed gaze shifts. *Developmental psychology* **50**, 100 (2014).
